# Molecular dynamics analysis of conserved water mediated inter-domain recognition of His667-Trp669 in human ceruloplasmin

**DOI:** 10.6026/97320630016209

**Published:** 2020-03-31

**Authors:** Bishnu Prasad Mukhopadhyay

**Affiliations:** 1 Department of Chemistry, National Institute of Technology-Durgapur, West Bengal, Durgapur - 713209, India

**Keywords:** Conserved water molecules, MD-simulation, Ceruloplasmin

## Abstract

The human ceruloplasmin (hCP) is the copper containing ferroxidase enzyme with multifunctional activities (NO-oxidase, NO2-synthase,oxidation of neurotransmitters including antioxidants). Therefore, it is of interest to probe the multi-domain hCP using moleculardynamics simulation. Results explain the role played by several conserved water centers in the intra and inter-domain recognition throughH-bond interaction with the interacting residues. We observed seventeen conserved water centers in the inter-domain recognition. Weshow that five invariant water centers W13, W14, W18, W23 and W26 connect the Domain 5 to Domain 4 (D5…W…W4). We also show thatfive other water centers W19, W20, W27, W30 and W31 connects the Domain 5 to Domain 6 (D5…W…W6) that is unique in the simulatedform. The W7 and W32 water centers are involved in the D1…W…W6 recognition. This is important for the water-mediated interaction ofGlu1032 to the trinuclear copper cluster present at the interface between these domains. The involvement of W10 water center in theD3…W10…D4 recognition through Gln552…W10…His667 H-bond interaction is critical in the complexation of CP with myeloperoxidase(Mpo). These observations provide insights to the molecular recognition of hCP with other biomolecules in the system.

## Background

Ceruloplasmin (hCP) is a copper containing ferroxidase enzyme. Beside the antioxidant properties of enzyme it also shows different activities e.g., oxidation of biogenic monoamines, NO-oxidase, NO2-synthase [1],glutathione-linked peroxidase [2] and able to prevent oxidative damage to protein, DNA and lipids [3] etc, however in most of the cases the reaction mechanisms are still unknown [4]. The X-ray structures have shown the presence of a trinuclear copper cluster and three mononuclear copper centers within the 3D-arrangement of six domains in the enzyme. Further MD-simulation studies have also indicated the role of conserved water molecules in the interaction with trinuclear and mononuclear copper centers of CP [5,6].Nevertheless the multifunctional enzyme need to have a well-defined and organized structure in order to maintain the functionality in physiological system, though some degree of flexibility of the domains and their recognition are required for catalytic activity or interaction with other macromolecular system. The structural and functional role of conserved water molecules and their endowment towards the intra and inter-domain recognition of proteins or metalloenzymes are well known [7,8]. However due to complexity in the structure of CP, the role of conserved water molecules in inter-domain recognition, hence their influence to the stability of overall structure or function is still not clear due to the limitations of resolution in the available crystal structures. Until now only one complex structure of CP with myeloperoxidase (the protein (Mpo) which is involved during inflammation) was solved at 4.69Å resolution by small angle X-ray diffraction method (PDB Id 4EJX) [9]. Nevertheless the coupling between acidic and basic residues (of different domains) through conserved water molecules is also thought to be an important aspect for intra/inter-domain stabilization, moreover in few cases those water centers may also participate in the redox coupling or proton exchange reaction.MD-simulation studies have provided the key insights into the importance of conserved water molecules in the intra/inter-domain recognition and their influence to the stability of CP structure. Furthermore, investigation of conserved water mediated recognition between Glu552 to His667, alongwith the conformational dynamics of His667 and Trp669 have also been made because of their importance in the complexation of ceruloplasmin with macromolecule myeloperoxidase (Mpo).

## Materials and Methods:

The PDB structure Id: 2J5W of CP[10] having 2.8Å resolution was used for MD- simulation studies . In the asymmetric unit, few metal ions, small organic molecules and 341 number of water molecules were present along with the ceruloplasmin molecule. The numbering scheme for copper ions, amino acid residues, and water molecules were followed in according to 2J5W crystal structure.

## Structure preparation:

 A)(i) O_2_ - bound hCP (from 2J5W PDB-structure):

The N-acetyl-D-glucosamine (NAG) molecules, an oxygen atom near to Cu3049, glycerol molecules, Ca^2+^ ion and one extra Cu^2+^ ion (which were incorporated in the crystal during crystallization) were removed from the 2J5W-PDB structure. The missing residues at the sequences 476-482 (Tyr-Asn-Pro-Gln-Ser-Arg-Ser), 885-889 (Tyr-Leu-Lys-Val-Phe) and 1042-1046 (Asp-Thr-Lys-Ser-Gly) were successively added to protein structure. The six integral copper atoms of trinuclear cluster and T1 mononuclear centers alongwith the O_2_ molecule was kept fixed at their respective crystallographic positions.Successive energy minimization of the structure was followed by steepest descent (1000 steps) and conjugate gradient (2000 steps) methods. Superimposing it on 2J5W crystal structure checked the final structure of protein, stereochemical arrangements of the residues were verified using Ramachandran plot.

(ii) O_2_ -bound hCP (from 4ENZ PDB-structure) [9] Superimposition between the 2J5W and 4ENZ crystal structures has shown the RMSD value of 0.34Å.The 4ENZ crystal structure (having 2.6 Å resolution) of CP contains only 82 number of water molecules, so we have also compared the water molecular position in both the PDB structures.The N-acetyl-D-glucosamine (NAG) molecules, glycerol molecules (and the other ions which were incorporated in the crystal during crystallization) were removed from the 4ENZ-PDB structure. The missing residues at the different sequences were successively added to protein structure. All the copper atoms of trinuclear cluster and T1 mononuclear centers and O_2_ molecule were kept fixed at the respective crystallographic positions. Successive energy minimization of the structure was done by steepest descent (1000 steps) and conjugate gradient (2000 steps) methods. Superimposing it on 4ENZ crystal structure checked the final structure of protein, and stereochemical arrangements of the residues were verified using Ramachandran plot.

## B) Apo structure of CP:

The six copper atoms and O_2_ - molecule were removed from the final energy minimized modelled structure of CP (built from the 2J5W-PDB structure). Then energy minimization was followed for removing the steric clashes, abnormal bond lengths and angles. Finally, the stereochemical arrangement of all the residues was checked again by Ramachandran plot. The standard deviation of the protein backbone between the simulated structures of 2J5W and Apo form of hCP was 0.08Å Identification of conserved water molecules. The 3DSS server [11] and Swiss PDB viewer program [12] were used to find out the conserved water molecules among the MD simulated and X-ray structures. The 2J5W PDB structure [13] was taken as reference and the MD- simulated structures at different time of simulation were successively superimposed on it. The cut-off distance between the pairs of superposed water molecules was taken to be 1.8 Å and only those were considered which have at least one hydrogen bond with the protein residue [14,15]. When a water molecule is found at a particular position (or within 1.8 Å) in the X-ray structures of a macromolecule or has high residential frequency (∼98-100%) at that site during simulation then it considered as static conserved water molecule (site), on the contrary when that water site in the X-ray structure was occupied by different water molecules at different time of simulation then that hydrophilic site is defined as dynamic conserved water center.

## Molecular dynamics (MD) simulation:

**Figure 1:**
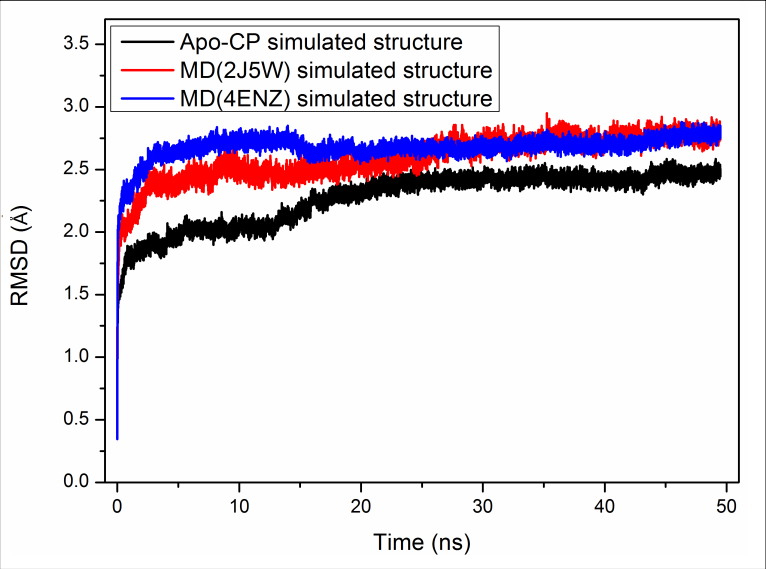
The root mean square deviation (RMSD) curves of hCP simulated structures. The apo-form is shown in black, 4ENZ and 2J5W simulated structures are shown in blue and red colour respectively.

Molecular dynamics simulation of both the O_2_-bound CP structures (2J5W and 4ENZ) and apo-form of ceruloplasmin structure were performed by NAMD v.2.6 [16,17] with CHARMM36 force field [18-20]. For O_2_-bound CP structure, the charges for copper atoms (Cu3046: 0.7937, Cu3047: 1.4304, Cu3048:1.4957, Cu3049: 1.4158, Cu3051: 0.7108, Cu3052:1.0425) and oxygen molecule (O1:-0.5084, O_2_:-0.5309) were obtained from our previous studies and they were successively added to the respective copper and oxygen atoms of O_2_ molecule [5] in both the 2J5W and 4ENZ structures. Then both the apo and O_2_-bound CP structures were converted to Protein Structure File (PSF) by Automatic PSF Generation Plug-in within VMD program v. 1.9.2 [21]. All the crystal water molecules, 341 in 2J5W and 82 in 4ENZ were added to the respective structures. In apo structure water molecules of the 2J5W structure were added accordingly. Then all these water molecules of the structures were converted to TIP3P water model [22]. Then adding appropriate number of sodium and chloride ions neutralized each system. Subsequent energy minimizations of the structures were performed by conjugate gradient method. The process was conducted in two successive stages; initial energy minimization was performed for 1000 steps by fixing the backbone atoms, followed by a final minimization for 2000 steps were carried out for all atoms of the system to remove residual steric clashes. Then each energy minimized structure was simulated separately at 310 K temperature and 1atm pressure by Langevin dynamics [23] using periodic boundary condition. The Particle Mesh Ewald method was applied for full-electrostatics and the Nose-Hoover Langevin piston method used to control the pressure and dynamical properties of the barostat. Then for each structure (apo-form and the two O_2_-bound form of CP) water dynamics was performed for 2 ns by fixing the protein residues and allowing the water molecules to move freely. Then all-atom molecular dynamics simulations for 50ns were carried out separately for both the apo and O_2_-bound human ceruloplasmin (2J5W modeled) structures. Moreover, 50ns MD-simulation of 4ENZ-modeled structure was also done. Atomic coordinates were recorded at every 2 ps for analysis. For each simulated structure, root mean square deviation (RMSD) of MD structures were calculated (by taking the X-ray structure as reference molecule) using RMSD trajectory tool in VMD (Figure 1).

## Results and discussion:

**Figure 2:**
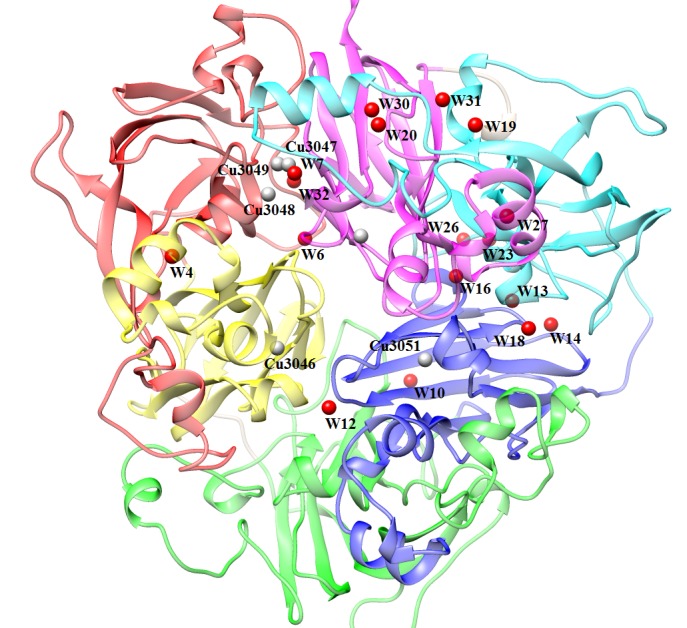
The conserved water molecules involved in the inter-domain recognition of ceruloplasmin. The domain 1 is shown in red, domain 2 in yellow, domain 3 in green, domain 4 in blue, domain 5 in cyan and domain 6 is shown in magenta colour. Red and grey spheres show the water molecules and copper atoms.

The structure of ceruloplasmin is mainly buildup with the six domains (D1-D6) having sequences: 1-192 (D1), 193-340 (D2), 347-553(D3), 554-703 (D4), 704-884(D5) and 891-1040 (D6) [24]. Several water molecules are observed to involve in the intra and inter-domain recognition in both the X-ray and MD-simulated structures of CP through H-bond interaction with the residues. Almost thirty-four number of water molecules are found to be conserved in both the X-ray and MD-simulated structures of 2J5W PDB-structure. Among these, seventeen number of water centers are played role in the stabilization of intra-domain residues whereas the other seventeen centers are involve to inter-domain recognition which have given in (Table 1). The occupation frequencies (O.F.) of those water sites are also included in that table. The conserved water centers that are involved in the inter-domain recognition have shown in (Figure 2). The interaction of conserved water centers with the different residues in the X-ray and MD-simulated structures of 2J5W are given in (Table 2).

## Conserve water molecules in intra-domain recognition:

Among the seventeen conserved water molecules (Table s1-Table 2), three water centers (W1, W2 and W3) are observed to interact with the residues of D1, W5 interacts with D2, two water centers (W8 and W9) to D3, three water centers (W11, W15 and W17) recognize D4, four water centers (W21, W22, W24 and W25) are interacting with D5 and the other four water centers (W28, W29, W33 and W34) have interact with D6 domain. During simulation of CP the static or dynamic character of the conserved water centers have been mentioned in (Table 1). Fifteen water centers (W1, W2, W3, W5, W8, W9, W11, W15, W21, W24, W25, W28, W29, W33 and W34) are observed to have ∼100% occupation frequency (O.F.) and the rest other (W17 and W22) have ∼95%. Stabilization of the intra-domain residues through conserved water (W1, W3, W8, W26) mediated salt-bridge interaction (acidic…water…basic) have also been observed in the simulated structures of 2J5W (Table S2), however some of them were not found in its crystal structure. Superimposition of 4ENZ crystal structure on the 2J5W crystal structure has also revealed the presence of eight invariant water molecules at the W2, W9, W21, W24, W25, W28, W29 and W33 sites (Table 1). The interactions of water molecules with the residues are almost found to be same in the crystal and simulated structures of 2J5W. Moreover, simulation studies of apo-ceruloplasmin structure have also revealed the presence of thirteen to fourteen static conserved water centers having ∼ 100% O.F. (Table S1) and they were also observed to be static in the 2J5W crystal and simulated structures with 100% O.F. Compiling all these results it may be presumed that at least the four invariant water centers W2 (W2040), W5 (W2066), W24 (W2270) and W29 (W2300) may play role in the structural stabilization of the respective D1, D2, D5 and D6 domains.

## Conserve water molecules in inter-domain recognition:

In the 2J5W-simulated structure, at least seventeen conserve water molecules (Table 1) are observed to involve in the inter-domain recognition through H-bond interaction of the residues of different domains (Table 2). Among these conserve water centers, ten are found to be static having ∼100% O.F. and other seven sites are dynamic in nature though their residential frequencies are observed to be high. Inter-domain (D) recognition of the residues by H- bond interactions through the conserved water centers: D1…W4…D2, D2…W6…D6, D4…W14…D5, D5…W19…D6, D5…W20…D6, D5…W27…D6, D5…W30…D6 and D1…W32…D6 are observed in both the X-ray and simulated structures of 2J5W (Table 2). However, stabilization of inter-domains by H-bonding interaction through conserved water centers like D1…W7…D6, D3…W10…D4, D2…W12…D4, D4…W13…D5, D4…W16…D6, D4…W18…D5, D4…W23…D5, D4…W26…D5 and D5…W31…D6 have been observed only in the simulated structures. Nevertheless the recognition between the domain 1 and domain 6 through two conserved water W7 and W32 center is thought to be important because the trinuclear copper cluster is situated at the interface between these two doamins. The W7 water molecule seems to connect the Glu1032 to that copper cluster (Glu1032…W7…Cu-cluster) which may be important for water mediated electron transfer process in CP[5,25]. The W10 water center has stabilized the His667 rotamer, which might be important for complexation of CP with Mpo. Nonetheless in the simulated structure of 2J5W several acidic and basic residues of the helix and loops (present at the surface of different domains in protein) have stabilized by conserved water mediated salt- bridge (Table S2) interaction: D1…W4…D2 (Glu207 (OE2)…W4…Lys50(NZ)), D4…W14…D5(Asp671(OD1)…W14… Arg845(NH2)), D4…W23…D5(Arg652 (NH2)…W23…Glu844(OE1)), D4…W26…D5 (Arg652(NH1)… W26…Glu844(OE2)), D5…W31…D6(Glu784 (OE2) …W31…Arg945(NH1)) though few of them are observed in its crystal structure. However in D5…W27…D6 and D1…W4…D2 inter-domain recognition in the 4ENZ-PDB structure are made through water mediated ionic interaction Lys761(NZ)…W1209…Glu906(OE2) and Lys50 (NZ)… W1214 …Gu207(OE1).

In the simulated structure of 2J5W, beside these water mediated coupling between the acidic and basic residues, some of those residues have also been stabilized by direct fork-fork type of salt-bridge between Glu844 and Arg652, where the OE1…NH2 and OE2…NH1 distances were varied from 2.7-3.1 and 2.74-3.2 Å. The Glu844 (OE1) residue also forms a water (W26) mediated salt-bridge with Arg882 (NH1). Similar type of fork-fork geometry has also been found in the salt-bridge between Glu784 and Arg 945, where the OE1…NH2 and OE2…NH1 distances were ranging from 2.7-3.2 and 2.63-3.23Å respectively. Moreover, fork-stick type of geometry has been observed in the salt-bridge Glu207(OE1)…Lys50(NZ) and Asp671(OD2)…Arg 845(NH1) where the distances were ranging from 2.5 to 3.0 and 2.51 to 2.8 Å respectively and these interactions were also observed in the 2J5W X-ray structure .

## Role of water molecule in conformational dynamics of His667 and Trp669

MD-simulation studies have also revealed the importance of a conserved or pseudo conserved water center (W10) in the recognition of D3…W10…D4 domains. The influence of that water molecule in the conformational dynamics of His667 and Trp669 residues has also been observed. In the 2J5W and 4ENZ PDB-structures these two residues are observed to stabilize by stacking interaction (with distance ∼4.1 Å), where the respective torsion angles χ^1^ (NB-CA-CB-CG) and χ^2^ (CA-CB-CG-CD2) of Trp669 in the structures are -62.78 and 84.73 in 2J5W, and -63.53 and 85.73 in 4ENZ. In stacking condition the χ^1^(NB-CA-CB-CG) and χ^2^(CA-CB-CG-ND1) values of His667 are -152.29 and 138.31in 2J5W, -145.97 and 136.80 in 4ENZ structure. However, the existence of another rotamer of His667 (χ^1^=63.04 and χ^2^=167.79) has also been indicated in the 4ENZ structure, which thus stabilized, by His667…W1202…Gln552 H-bond interaction. In both the crystal structures, Gln552 of domain 3 is found to stabilize by a water molecule of W10 site (W2126 in 2J5W and W1202 in 4ENZ structures) through H-bonds (∼2.99Å), which were given in (Table S1 and Table S2). Nevertheless, such stabilization mechanism of His667 rotamer has also been observed in the simulated structure of CP though there were some variations in the torsion angles.

**Figure 3:**
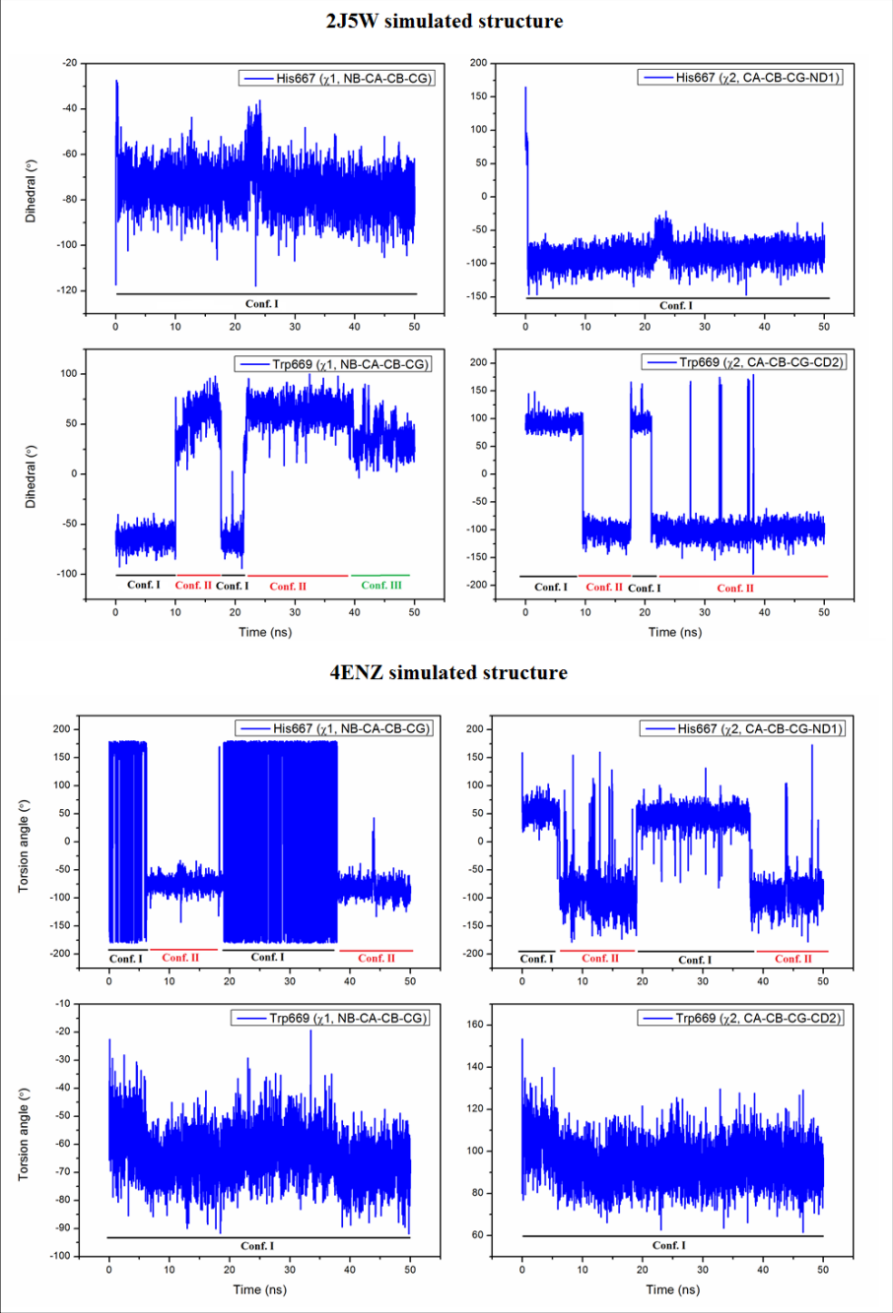
The variation of torsion angles (χ^1^ and χ^2^) with time (ns) of His667 and Trp669 in the 2J5W and 4ENZ simulated structures.

During simulation of 4ENZ structure, His667 shows two preferred conformations I and II, where the χ^1^ and χ^2^ values are ∼176° and 50° (for I), and ∼ -75° and -80° (for II). The rotamer I of His667 exists from 0 to 6.2 and 19.5 to 37.8ns, whereas the II-rotamer is exists from 6.25 to 19.45 and 37.83 to 50ns. The variation of torsion angles of that residue with time has shown in (Figure 3). From the initial stage, the conformation I of His667 is stabilized by stacking interaction with Trp669 upto 6.2ns. However, after adopting conformation II at ∼6.25ns the residue is stabilized by water mediated inter-domain D3…W10…D4 interaction through His667(NE2)…W10…Gln552(OE1) H-bonds. After 19.5ns, His667 is again revert back to conformation I, and the water molecule (W1202) is observed to migrate from that conserved site (W10) at ∼20ns. The occupation frequency of W10 water center is observed to be ∼40%. Again the imidazole residue seems to adopt conformation II at ∼37.83 which exists upto 50ns. However in the entire simulation period, the Trp669 stays almost at its position (conformation I), where the χ^1^ and χ^2^ values are ∼ -65° and 104° which are shown in (Figure 3).

During simulation of 2J5W structure, His667 is found to stabilize by Trp669 through π...π interaction upto 0.5ns, however after that period histidine adopts conformation II which exists upto ∼50ns (where the χ^1^ and χ^2^ values are ∼ -75° and -80°) and it is stabilized by Gln552 bound water molecule W2126 through His667 (NE)…W2126…Gln552(OE) H-bond interaction, where the His667(NE)…W2126 and Gln552…W2126 distances were varied from 2.86 to 3.11 and 2.65 to 2.98Å. Actually W2126 water molecule has occupied the conserved water site W10 with ∼100% O.F. During simulation of hCP, Trp669 shows different conformations, initially from 0 - 9.98ns, it stays almost at its initial position with conformation I (where the torsion angles χ^1^ and χ^2^ are ∼ -68° and 98°). But after ∼10ns the indole ring is parallel displaced from its initial position and adopts conformation II (χ^1^ and χ^2^ angles are ∼ 68° and -100°) which exists upto ∼17.5ns, where the residue was stabilized by H-bond interaction with water molecules (W…Trp669(NE)). The conformation II (of Trp669) has reappeared at ∼21.9ns and exist upto 7sim;40ns. After that period, tryptophan adopts conformation III (where the χ^1^ and χ^2^ values are ∼ 25° and -100°), where the indole ring lies almost perpendicular to previous conformation-I thus it stabilized by Trp669 (π)…water (W2175) interaction. The variations of torsion angles for Trp669 and His667 with time are shown in (Figure 3). In the two simulated structures variation of occupation frequency of Gln552 bound water molecule (W10) may arise due to lower number of water molecules in the asymmetric unit of 4ENZ crystal compared to 2J5W structure. In ceruloplasmin, several conserved water molecules are playing role in the inter-domain recognition and structural stabilization. The W7 water center is playing role in the interaction of Glu1032 to trinuclear copper cluster. It is interesting to observe the role of conserved water molecule in the dynamics of His667 and Trp669 residues which may be important for the interaction of CP with the macromolecule myeloperoxidase (Mpo). Possibly, nature of these conserved water centers and their interaction with the intra and inter-domain residues are also thought to be important for keeping the proper structural flexibility of that multifunctional enzyme and the recognition of hCP with other biomolecules.

## Conclusions:

Molecular dynamics analysis of the O_2_-bound ceruloplasmin structure show 34 conserved water sites. We observed that 17 centers are directly interacting and stabilizing the intra-domain residues through H-bonds. However, 17 other water centers are involved in the inter-domain recognition and are connected with the inter-domain residues through conserved water mediated H-bonds. The four invariant water molecules at the W2, W5, W24 and W29 sites are involved in the structural stabilization of ceruloplasmin. We report 10 conserved water centers involved in the inter-domain stabilization of domain 5 (D5). The 5 water centers W13, W14, W18, W23 and W26 are connected with domain 4 (D5…W…D4). Moreover, the 5 other water centers W19, W20, W27, W30 and W31 are involved in D5…W…D6 recognition. The W7 and W32 water centers connect the D1-domain to D6-domain through H-bonds. These water-mediated interactions (Glu1032…W7…Cu-cluster) are important to the electron transfer process of hCP as the trinuclear copper cluster is situated at the interface between domain 1 and 6 as described elsewhere [5]. The water molecule at the W10 center participates in the D3…W10…D4 recognition by Gln552…W10…His667 H-bond interaction, which stabilizes the complexation of CP with Myeloperoxidase (Mpo). This is interesting. The conserved water mediated interaction of the residues and their involvement to inter-domain stabilization have implication in the recognition biology of CP with other biomolecules.

## Figures and Tables

**Table 1:** The occupation of different water molecules at the conserved hydrophilic sites during MD- simulation of O2- bound ceruloplasmin structure.

S.No. of conserved water sites	Id No. of Crystal water molecules (PDB Id 2J5W)	Id No. of water molecules which are occupied in the conserved hydrophilic water sites during MD- simulation (of 2J5W PDB structure) at different time (ns)					Occupation frequency (%) / type of conserved water centers	Conserved water centers played role in the intra/ inter-domain (D) recognition during MD-simulation	Conserved hydrophilic positions occupied by water molecules in 4ENZ PDB structure
		10	20	30	40	50			
W1	W2005	W2003	W2005	W2003	W2065	W2065	100/ dynamic	Intra(D1)	
W2	W2040	W2040	W2040	W2040	W2040	W2040	100/ static	Intra (D1)	W1225
W3	W2043	W2043	W2001	W2048	W2048	W2076	100/ dynamic	Intra(D1)	
W4	W2054	W2054	W2054	W2054	W2054	W2054	100/ static	Inter	W1214
W5	W2066	W2066	W2066	W2066	W2066	W2066	100/ static	Intra(D2)	
W6	W2080	W2037	W2260	W2260	NA	W2162	93/ dynamic	Inter	
W7	W2090	W2331	W2331	W2331	W2331	W2331	100/ static	Inter	
W8	W2106	W2106	W2106	W2106	W2106	W2106	100/ static	Intra(D3)	
W9	W2115	W2115	W2115	W2115	W2115	W2115	100/ static	Intra(D3)	W1218
W10	W2126	W2126	W2126	W2126	W2126	W2126	100/ static	Inter	W1202
W11	W2148	NA	W2132	W2148	W2148	W2148	95/ dynamic	Intra(D4)	
W12	W2152	W2152	W2152	W2152	W2152	W2152	100/ static	Inter	
W13	W2154	W2154	W2154	W2154	W2154	W2154	100/ static	Inter	
W14	W2156	W2156	W2156	W2156	W2156	W2156	100/ static	Inter	
W15	W2160	W2257	WW2257	W2257	W2257	W2257	100/ static	Intra(D4)	
W16	W2167	W2318	W2318	W2318	W2079	W2079	100/ dynamic	Inter	
W17	W2168	NA	W2075	W2020	W2261	W2317	95/ dynamic	Intra(D4)	
W18	W2179	W2178	W2178	W2249	W2157	W2157	100/ dynamic	Inter	W1257
W19	W2197	W2233	W2197	W2197	W2197	W2197	100/ dynamic	Inter	
W20	W2199	W2199	W2199	W2199	NA	W2241	97/ dynamic	Inter	
W21	W2221	W2221	W2221	W2221	W2277	W2221	100/ dynamic	Intra(D5)	W1201
W22	W2244	W2244	W2244	W2244	NA	W2266	95/ dynamic	Intra(D5)	
W23	W2256	W2191	W2236	W2160	W2236	W2111	100/ dynamic	Inter	
W24	W2270	W2270	W2270	W2270	W2270	W2270	100/ static	Intra(D5)	W1226
W25	W2271	W2181	W2181	W2181	W2181	W2181	100/ static	Intra(D5)	W1232
W26	W2273	W2256	W2320	W2236	W2320	W2320	100/ dynamic	Inter	W1261
W27	W2279	W2279	W2279	W2279	W2279	W2279	100/ static	Inter	W1209
W28	W2286	W2286	W2286	W2286	W2286	W2286	100/ static	Intra(D6)	W1212
W29	W2300	W2300	W2300	W2300	W2300	W2300	100/ static	Intra(D6)	W1223
W30	W2302	W2302	W2302	W2302	NA	W2302	97/ static	Inter	
W31	W2303	W2231	W2231	W2231	W2231	W2231	100/ static	Inter	
W32	W2311	W2033	W2033	W2033	W2033	W2033	100/ static	Inter	
W33	W2316	W2316	W2316	W2316	W2316	W2316	100/ static	Intra(D6)	W1234
W34	W2321	W2322	W2322	W2322	W2322	W2189	100/ dynamic	Intra(D6)	

**Table 2:** Hydrogen bonding interaction of the residues with the different conserved hydrophilic water sites during the simulation of O2- bound ceruloplasmin and also in the X- ray structures (PDB Id 2J5W).

Conserved hydrophilic (water) sites		Residues interacts with the conserved water sites in the X- ray and MD- simulated structure during 50ns^*^		Interdomain (D) recognition by conserved water center (D…W…D)	
		Interact in the X- ray structure	Interact only during MD- simulation	X-ray structure	MD- structure
	W1		Lys23 (NZ), Glu22(OE1), Phe248(OB)		
	W2	Gly173(OB), Phe73(OB), Tyr53(NB)	Gly173(OB), Phe73(OB), Tyr53(NB)		
	W3	Leu186(OB)	Glu184(OD2) , Lys192(NZ),		
**	W4	Glu207(OE1), Lys50(NZ), Lys49(OB), Ser210(OG)	Glu207(OE1), Lys50(NZ), Lys49(OB), Ser210(OG)	D1…W4…D2	D1…W4…D2
	W5	Ser242(OG), Asn244(NB), Glu245(NB)	Ser203(OG),Ser242(OG), Asn244(NB), lu245(NB)		
	W6	Leu302(OB),Val996(OB), Ser994(OG)	Leu302(OB), Val996(OB), Ser994(OG)	D2…W6…D6	D2…W6…D6
	W7		Glu1032(OE1), Ala166(OB)		D1…W7…D6
	W8	Glu424(OE1),Arg420(NH2)	Glu408(OE1),Glu424(OE1), Arg420(NH2)		
	W9	Trp500(NE1), Tyr498(π), Asn467(OB), Ile456(OB)	Trp500(NE1), Tyr498(π), Asn467(OB), Ile456(OB)		
	W10	Gln552(OE1),Cys512(NB/OB), Val514(NB)	His667(NE2),Gln552(OE1), Cys512(NB/OB), Val514(NB)		D3…W10…D4
	W11	Ser603(OG), Asn605(NB), Phe562(OB), Gly606(NB)	Ser603(OG), Asn605(NB), Phe562(OB), Gly606(NB)		
	W12		Gln320(OE1), Asn321(NB), Ala630(OB)		D2…W12…D4
	W13	Phe641(OB) Asn644(OB)	Gly819(NB),Phe641(OB) Asn644(OB)		D4…W13…D5
**	W14	Asp671(OD2),Thr672(OG1) Gly643(OB) Arg845(NH2)	Asp671(OD2),Thr672(OG1) Gly643(OB),Arg845(NH2)	D4…W14…D5	D4…W14…D5
	W15	Arg652(NH1)	Trp669(NE), Arg652(NH1)		
	W16		Asp654(OD1),Gly1002(OB)		D4…W16…D6
	W17	Asn657(OB)	Thr662(OG),Asn657(OB)		
	W18	Asn677(OD1),Glu679(OE1) Asn677(ND2)	Gln866(OE1),Tyr861(OH), Glu679(OE1),Asn677(ND2)		D4…W18…D5
	W19	Asp725(OB/OD1),Arg945(NH2) Trp724(π)	Asp725(OB/OD1),Arg945(NH2) Trp724(π)	D5…W19…D6	D5…W19…D6
	W20	Glu733(OE1),Gln729(OB) Asn949(ND2)	Glu733(OE1),Gln729(OB) Asn949(ND2)	D5…W20…D6	D5…W20…D6
	W21	Gln767(NB)	Gln767(NB),Glu783(OE1/OE2) Val777(OB)		
	W22	His816(NE2),Trp840(NE) Ile815(OB)	Trp840(NE),Ile815(OB)		
**	W23	Glu844(OB/OE1)	Arg652(NH2),Glu844(OB/OE1)		D4…W23…D5
	W24	Gly873(OB),Ile788(OB) Val765(NB)	Gly873(OB),Ile788(OB)		
			Val765(NB)		
	W25	Leu870(NB),Leu874(OB) Ser862(NB)	Leu870(NB),Leu874(OB) Ser862(NB)		
**	W26	Glu844(OE1)	Glu844(OE1),Arg652(NH2)Arg882(NH1),Glu844(OE1)		D4…W26…D5
	W27	Lys761(OB),Glu906(OE2) Ser909(OG),Asn915(ND2)	Lys761(OB),Glu906(OE2) Ser909(OG),Asn915(ND2)	D5…W27…D6	D5…W27…D6
	W28	Glu906(OE2/OB),Asn915(ND2)	Glu906(OE2/OB),Asn915(ND2)		
	W29	Leu900(OB),Ala941(OB) Gly944(NB)	Leu900(OB),Ala941(OB) Gly944(NB)		
	W30	Gln729(OE1/OB),Trp732(NB) Phe947(OB)	Gln729(OE1/OB),Trp732(NB) Phe947(OB)	D5…W30…D6	D5…W30…D6
**	W31	Arg945(NH1),Asn949(OB) Gln951(NB)	Glu784(OE2),Arg945(NH1) Asn949(OB),Gln951(NB)		D5…W31…D6
	W32	Gln146(OB),Arg158(NH2) Ser993(OG)	Gln146(OB),Arg158(NH2) Ser993(OG)	D1…W32…D6	D1…W32…D6
	W33	Asp995(OD2),Phe979(OB)	His982(OB),Asp995(OD2) Phe979(OB)		
	W34	Ser983(OB),Phe1010(OB)	Ser983(OB),Phe1010(OB)		
*Residue to water distances which are within 1.90Å to 3.50Å (during simulation) are included in this table. **Conserved water molecules/centers which are involved in the inter-domain recognition through the interaction of acidic (Aspartic, Glutamic) and basic (Lysine, Arginine) residues.

**Table S1:** Conserved water molecules in the MD simulated structures of Apo form of CP

Conserved Water positions	2J5W X-ray Structure	MD-simulated structures at different time (ns)				
		10	20	30	40	50
WA1	W2022	W2022	W 2022	W2022	W 2022	W 2022
WA2	W 2036	W2036	W2078	W2080	W2068	W2168
WA3	W 2040	W2040	W2040	W2040	W2040	W2040
WA4	W 2054	W2054	W2054	W2054	W2095	W2095
WA5	W2066	W2066	W2066	W2066	W2066	W2066
WA6	W2080	W2036	W2020	W2317	W2168	W2080
WA7	W2085	W2085	W2085	W2085	W2085	W2085
WA8	W2102	NA	W2102	W2102	W2102	W2102
WA9	W 2103	W 2106	W 2091	W 2119	NA	W2034
WA10	W2116	W2116	W2116	W2116	W2116	W2116
WA11	W2127	W2127	W2127	W2127	W2127	W2127
WA12	W2154	W2157	W2183	W2183	W2183	W2183
WA13	W2159	W2160	W2134	W2274	W2134	W2134
WA14	W2184	NA	W2184	W2205	W2065	W2065
WA15	W2221	W2221	W2229	W2229	W2229	W2229
WA16	W2256	W2256	W2256	NA	W2256	W2256
WA17	W2261	W2235	W2165	W2312	W2312	W2276
WA18	W2262	W2181	NA	W2181	W2181	W2181
WA19	W2270	W2270	W2270	W2270	W2270	W2270
WA20	W2271	W2271	W2271	W2271	W2271	W2271
WA21	W2272	W2272	W2272	W2272	W2272	W2272
WA22	W2300	W2300	W2300	W2300	W2300	W2300
WA23	W2302	W2302	W2302	W2302	W2302	W2302
WA24	W 2311	W 2033	W 2033	W 2033	W 2033	W 2033
WA25	W2321	W2321	W2321	W2321	W2321	W2321

**Table S2:** Intra and Inter- domain recognition through direct and conserved water mediated salt bridge interaction of the residues (Acid…Water…Base) in the X-ray and MD-simulated (2J5W) structures of human Ceruloplasmin. All the distances are given in Å unit.

Recognition through conserved water mediated salt bridge interaction (Acid (A)…Water(W)…Basic (B))		H-bonding distances (Å) of the acidic and basic residues form the water molecules			
		X-ray structure (PDB Id 2J5W)		Ranges of distances for water mediated salt bridge interaction during MD- simulation	
		A…B	A…W…B	A…B	A…W…B
Intra domain	Glu22(OE1)…W1…Lys23(NZ)	-	-	-	2.58-3.01,2.52-3.05
	Glu184(OD2)…W3…Lys192(NZ)	-	-	-	2.72-3.34,2.81-3.50
	Glu424(OE1)…W8…Arg420(NH2)	-	5.17,3.15	-	2.53-3.32,3.0-3.20
	Glu408(OE1)…W8…Arg420(NH2)	-	5.17,3.15	-	2.70-2.91,3.0-3.21
	Glu844(OE1)…W26…Arg882(NH1)	-	5.26,2.83	-	2.70-3.10,2.91-3.33
Inter domain	Glu207(OE2)…W4…Lys(NZ)	2.56	2.48,2.76	2.49-3.02	2.51-3.03,2.71-3.11
	Asp671(OD1)…W14…Arg845(NH2)	3.04	2.65,3.34	2.51-2.84	2.81-3.52,3.81-4.02
	Glu844(OE1)…W23…Arg652(NH2)	3.99	4.89,3.82	2.70-3.12	3.10-3.71,2.61-3.13
	Glu844(OE2)…W26…Arg652(NH1)	-	2.56,5.19	2.74-3.12	2.73-3.12,3.0-3.42
	Glu784(OE2)…W31…Arg945(NH1)	-	3.78,3.25	2.70-3.10	2.61-2.82,3.0-3.42
